# Assessing nuclear versus mitochondrial cell-free DNA (cfDNA) by qRT-PCR and droplet digital PCR using a piglet model of perinatal asphyxia

**DOI:** 10.1007/s11033-022-08135-0

**Published:** 2022-12-13

**Authors:** Marie Bitenc, Benedicte Grebstad Tune, Maria Melheim, Monica Atneosen-Åsegg, Xiaoran Lai, Polona Rajar, Rønnaug Solberg, Lars Oliver Baumbusch

**Affiliations:** 1 Department of Pediatric Research, Division of Paediatric and Adolescent Medicine, Oslo University Hospital Rikshospitalet, Postbox 4950, 0424 Nydalen, Oslo Norway; 2 Institute of Clinical Medicine, University of Oslo, Oslo, Norway; 3 Oslo Centre for Biostatistics and Epidemiology, Faculty of Medicine, University of Oslo, Oslo, Norway; 4 Department of Neonatal Intensive Care, Division of Paediatric and Adolescent Medicine, Oslo University Hospital Ullevål, Oslo, Norway; 5 Institute of Oral Biology, University of Oslo, Oslo, Norway; 6 Department of Pediatrics, Vestfold Hospital Trust, Tønsberg, Norway

**Keywords:** Cell-free DNA, Digital PCR, Mitochondrial cfDNA, Nuclear cfDNA, Perinatal asphyxia, qRT-PCR

## Abstract

**Background:**

Since the discovery more than half a century ago, cell-free DNA (cfDNA) has become an attractive objective in multiple diagnostic, prognostic, and monitoring settings. However, despite the increasing number of cfDNA applications in liquid biopsies, we still lack a comprehensive understanding of the nature of cfDNA including optimal assessment. In the presented study, we continued testing and validation of common techniques for cfDNA extraction and quantification (qRT-PCR or droplet digital PCR) of nuclear- and mitochondrial cfDNA (ncfDNA and mtcfDNA) in blood, using a piglet model of perinatal asphyxia to determine potential temporal and quantitative changes at the levels of cfDNA.

**Methods and Results:**

Newborn piglets (n = 19) were either exposed to hypoxia (n = 11) or were part of the sham-operated control group (n = 8). Blood samples were collected at baseline (= start) and at the end of hypoxia or at 40–45 min for the sham-operated control group. Applying the qRT-PCR method, ncfDNA concentrations in piglets exposed to hypoxia revealed an increasing trend from 7.1 ng/ml to 9.5 ng/ml for *HK2* (hexokinase 2) and from 4.6 ng/ml to 7.9 ng/ml for β-globulin, respectively, whereas the control animals showed a more balanced profile. Furthermore, median levels of mtcfDNA were much higher in comparison to ncfDNA, but without significant differences between intervention versus the control group.

**Conclusions:**

Both, qRT-PCR and the droplet digital PCR technique identified overall similar patterns for the concentration changes of cfDNA; but, the more sensitive digital PCR methodology might be required to identify minimal responses.

**Supplementary Information:**

The online version contains supplementary material available at 10.1007/s11033-022-08135-0.

## Introduction

Changes in the amount of cell-free DNA (cfDNA) in peripheral blood have been associated with many different conditions and diseases leading to the establishment of liquid biopsies for a range of medical applications [1–5]. However, many questions remain about the composition and molecular mechanisms of cfDNA generation and distribution [6, 7]. Initially, elevated quantities of cfDNA have been observed for a variety of severe diseases and conditions, including malignancies, autoimmune diseases, trauma, burn injuries, sepsis, stroke, myocardial infarction, and organ transplantations [8–10]. The assumption of a disease-dependent spread of DNA fragments is counteracted by the observation of base-line levels of cfDNA detected in the blood circulation of healthy individuals, with age, sex, hormonal, biorhythmic, and other dependencies [11]. Further, cfDNA fragments are not only circulating in blood, but can also be found in other body fluids, like saliva, urine, or cerebrospinal fluid [12–14].

From a molecular biology perspective, cfDNA fragments are part of the comprehensive cell-free nucleic acid (cfNA) family, presented in the form of vesicle bound- and non-vesicle bound cfNAs (e.g., exosomes, microvesicles, or apoptotic bodies) and cfNAs macromolecular complexes (e.g., nucleosomes, virtosomes, neutrophil extracellular traps (NETs), or high/low-density lipoproteins) [15, 16]. CfNAs are commonly defined as highly fragmented (80–200 bp), single- or double-stranded molecules with a molecular weight in the range of 0.18–21 kB [3]. These components may be endogenous- or exogenous, originating from nuclear or mitochondrial DNA, besides cell-free RNAs [14, 17, 18]. For the molecular mechanisms of cfDNA generation and distribution, two distinct principles have been proposed: a passive release through cell decomposition, e.g., apoptosis, necrosis, or physical damage of the cell, or an active mechanism via vesicles and lipoprotein-nucleotide complexes, facilitating local or long-distance cell-to-cell communication [3, 15, 19, 20].

**Fig. 1:**
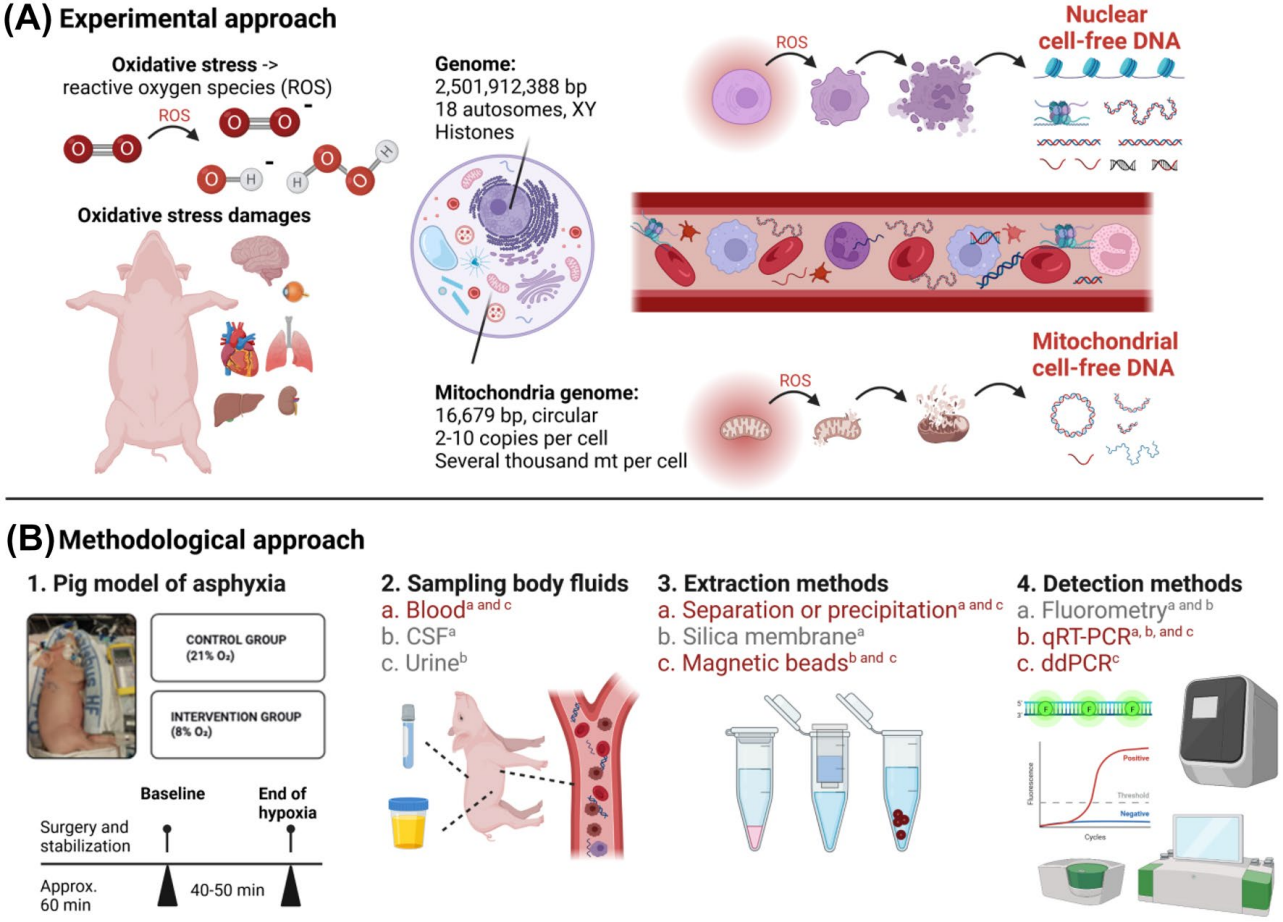
Experimental and methodological approaches to investigate the nature of cfDNA using a piglet model of perinatal asphyxia. **A**. Assessment of changes in the amount of cfDNA in various body fluids has become an interesting tool for many diseases and conditions, including cancer, prematurity, or hypoxia. We have previously postulated [12, 13], that the observed variations in cfDNA might be provoked by reactive oxygen species (ROS) damaging various cell structures. Consequently, fragments of both, nuclear and mitochondrial cfDNA (ncfDNA and mtcfDNA, respectively) may be released into the blood stream. **B**. Using a piglet model of perinatal asphyxia, the animals were distributed either into a cohort exposed to hypoxia or to the sham-operated control group. Blood samples were collected at baseline (= start) and at the end of hypoxia or at 40–45 min for the sham-operated control group, respectively. Additional body liquids, different extraction and quantification methods have previously been tested and compared (a = [12] and b = [13]). C = here in this study (indicted in red), the quantities of nuclear (at two loci) and mitochondrial cfDNAs in blood samples were measured by qRT-PCR and ddPCR. (Abbreviations: cerebrospinal fluid, CSF; droplet digital PCR, ddPCR; mitochondria, mt; and quantitative real-time PCR, qRT-PCR. Figure created using BioRender.com)

We have previously proposed a cellular mechanism of cfDNA production and discharge via the generation of reactive oxygen species (ROS) in both, the nucleus and in the mitochondria [12, 13]. Nuclear and mitochondrial genomes are principally different, in terms of biological tasks, size, copy number, and organization, with striking consequences for cfDNA determination (Fig. 1.). Consequently, measuring only one genomic locus or solely ncfDNA or mtcfDNA may not be sufficient to provide comprehensive information about the principal changes ongoing in an organism [21–23].

With an incidence of 1.5–2.5 per 1000 live births in developed countries, and even 10–20 fold higher in low- and middle-income countries, perinatal asphyxia is still one of the major causes of early neonatal mortality and morbidity [24, 25]. Many different markers have been proposed to estimate the oxidative stress reactions and cell damages in its pathophysiological background; however, the key regulatory elements are still not identified [12]. Studies have proposed that cfDNA might be an early indicator of perinatal asphyxia [12, 13]. Moreover, Tuaeva et al*.*, 2008 observed that premature birth is associated with increased cfDNA concentrations in the blood of neonates [26]. These findings built the basis to investigate circulating cfNAs as a potential biomarker in the diagnostics of the damages caused by perinatal asphyxia [12, 13]. But, the analysis of cfDNA is challenging, including the best choice of extraction processes, quantification techniques, and applications for the assessment of cfNAs [11, 27–30]. Optimization of pre- (e.g., sample collection, handling, transportation, storage, extraction, and preparation) and post-analytical procedures are crucial to maximize the performance [27–30]. The challenges measuring cfNAs are mainly due to genomic DNA contamination, low concentration, high fragmentation, post-sampling cell degradation, short half-life, and limitations of available detection technologies. The concentration of cfDNA has been investigated by several techniques, including the analysis of agarose or polyacrylamide gels, spectrophotometer, fluorometry with SYBR Green, SYBR Gold, Hoechst dye 33,342 or PicoGreen, capillary zone electrophoresis, quantitative real-time PCR (qRT-PCR), or digital PCR [17, 31, 32].

Here, we present the newest efforts of our consecutive testing of various cfDNA assessment applications (qRT-PCR or droplet digital PCR (ddPCR)) for ncfDNA and mtcfDNA analyses (Fig. 1.). Using a piglet model of perinatal asphyxia, cfDNA was previously examined in a range of body fluids (blood, urine, and cerebrospinal fluid) [12, 13]. The aim of this study was to estimate, if genomic loci have an influence on the content of ncfDNA and to investigate the relationship of ncfDNA in comparison to changes in the quantity of mtcfDNA, to better understand the general methodological and biological features of cfDNA. To this end, we compared samples of two treatment regimes (hypoxia versus sham-operated control samples). Further, the methodological approach was to evaluate the performance of the common qRT-PCT method in comparison to the state-of-the-art technique of ddPCR.

## Material and methods

### Study design

A subset of n = 19 newborn (age 12–36 h) Noroc piglets were included in the study, as part of a larger study of N = 42 piglets, with other treatment regimes, not relevant for the purpose of this study. The temperature of the piglets was continuously measured and maintained at 38.5–39.5 °C. Piglets were anesthetized with a dose of pentobarbital (15 mg/kg) intravenously through a cannula in an ear vein. They were orally intubated, ventilated, and surgically prepared, as described by Benterud et al*.*, 2015 [33]. Intravenous infusion of fentanyl (0.05 mg/kg/hour) and Benelyte® was constantly given throughout the procedure. In case of severe shivering, increase in blood pressure, high pulse, or increased tone, additional bolus of pentobarbital (2.5 mg/kg) was provided.

In this study design, animals were exposed to severe global hypoxia or treated as sham- operated controls. Piglets were stabilized for one hour before proceeding with the experimental protocol. Piglets in the intervention group (n = 11) were exposed to global hypoxia for a mean period of about 49 min and until severe drop in blood pressure and thereby perfusion and SaO_2_, as typically observed for perinatal asphyxia. Hypoxia was introduced by ventilation with 8% O_2_ in N_2_ until base excess (BE) reached − 20 mM or the mean arterial blood pressure (MABP) decreased to 20 mmHg. Animals in the sham-operated control group (n = 8) received 21% O_2_ throughout the entire experiment (no exposure to hypoxia) but otherwise the same procedures.

All the piglets were observed for 9.5 h after the end of the hypoxia exposure. Blood gases, pulse, oxygen saturation, blood pressure, and temperature were continuously assessed. Moreover, the anesthesia and analgesia were monitored continuously.

Blood samples were drawn at baseline (= at the start and thus, prior to the exposure to hypoxia) and for the intervention group at post-intervention (= end of hypoxia). For the sham-operated control group, the second blood samples was taken after approximately 40–45 min, which is about the equivalent to an average time period of hypoxia in the intervention group, based on previous experiments [33]. The piglets were finally euthanized with an intravenously provided overdose of pentobarbital (150 mg/kg).

Some of the piglets did not tolerate the effects of the harsh treatment until the scheduled end of the experiment; consequently, for these animals not all samples could be collected for the analysis (Supplementary Table 1.). Additionally, abnormalities in some harvested organs were observed for two piglets (Supplementary Table 1.). The extreme outcomes and tissue abnormalities may reflect changes and thus, we decided to include all possibly available samples and exclude only outliers identified by the inter quartile range (IQR) method (see statistics section).

### Blood samples

Approximately 2 ml of blood were collected and transferred into a 2 ml tube with EDTA and centrifuged at 1,700 × g for 10 min at 4 °C. After the centrifugation, two times 0.5 ml plasma containing the cfDNA were transferred into new Eppendorf tubes, snap-frozen in liquid nitrogen, and stored at − 80 °C (Supplementary Fig. 1.). The second sample was collected and stored for prospective further analyses.

### The isolation of cfDNA

CfDNA was initially extracted from plasma using the Mag-Bind cfDNA Kit (Omega Biotek), following the recommendations of the KingFisher Duo Prime Purification System (Thermo Fisher Scientific). Due to the low input starting volume of 450 μl, some modifications to the protocol were necessary to ensure high enough cfDNA concentrations for down-stream applications. Collect count in the elution step was increased from three to four and the final elution volume was set to 80 μl. Phosphate-buffered saline (PBS) was added to three of the samples (piglet nr. 21 at baseline and piglet nr. 20 and nr. 25 at the end of hypoxia) to equalize the settings of the starting plasma volume for cfDNA extraction. Samples of extracted cfDNA were stored at − 80 °C for up to one week until being further processed.

### Yield of cfDNA and determination of the fragment size

We first estimated the yield and fragment size of extracted cfDNA from pooled plasma samples using both, the original and modified isolation protocol. Further, we tested the effect of freezing and thawing cycles of the extracted cfDNA samples on their fragment lengths using three aliquots. Each of the aliquots was frozen at − 80 °C for at least 60 min, thawed, and proceeded with the Agilent cfDNA Screen Tape (Agilent Technologies) according to the manufacturer's protocol on the Agilent 4200 TapeStation (Agilent Technologies). The process of freezing and thawing was repeated five times (Supplementary Table 2.).

### Sampling and processing of cfDNA

Two different quantification approaches were tested and applied to all samples of extracted cfDNA from piglet plasma with primers for both, ncfDNA and mtcfDNA: qRT-PCR and ddPCR. For the ddPCR method, we additionally assessed the amount of cfDNA in unprocessed plasma samples, without prior cfDNA extraction.

### Standard curve

Standard samples with known DNA concentrations were made using a porcine DNA stock (0.53 μg/μl) from normal tissue (AMSBIO). Concentration in the standard sample with the highest value was verified using the Qubit dsDNA high-sensitivity (HS) (Thermo Fisher Scientific Inc.) and then diluted 1:10 in the elution buffer.

### Quantitative RT-PCR reactions and conditions

Three different primer pairs were used to quantify cfDNA by qRT-PCR. The primers were designed using Primer Express 3.0.1 (Applied Biosystems) [13]. The settings recommendations of Applied Biosystems (Tm 58–60 °C, GC-content 40–70%, primer length 18–25 bp, < 2 difference between primer pairs, 2–3/5 G or C at the 5′end, maximum of 2/5 G or C at the 3′end, < 4 continuous G, C, A, T, secondary structure was also checked for hairpins or other cross-connections) were used and the primer efficiency was tested. For ncfDNA: *HK2* (hexokinase 2): FP: 5′-GTTCCTGGCTCTGGATCTTGG-3′ (Tm: 59.7 °C), RP 5′-GCCACTGCCTCGCATGA-3′ (Tm: 59.0 °C), amplicon length 133 bp and β-globulin locus: FP: 5′-GCAAGCTGCTGGTTGTCTAC-3′ (Tm: 54.8 °C), RP: 5′-GTCACTGAAGGACTGGAGCA-3′ (Tm: 54.9 °C), amplicon length: 134 bp (β-globulin was only used for quantification by the qRT-PCR method but not by the ddPCR method). For mtcfDNA, *NADH6* (NADH dehydrogenase subunit 6): FP: 5′-TCACCCTCAATGACGAACAAGA-3′ (Tm: 59.2 °C), RP: 5′-TAGGGCTCAGGCGTTTGTGTA-3′ (Tm: 59.4 °C), amplicon length 140 bp. Reaction mixtures contained 4 μl DNA standards, sample, or a negative control, 0.8 μl primer mix (400 nM), 10 µl SYBR Green mix, and 5.2 μl nuclease-free water to a final volume of 20 μl. The qRT-PCR program was set to an initial activation step at 50 °C for 2 min and 95 °C for 10 min, followed by 40 cycles of denaturation step at 95 °C for 15 s and continued with annealing and elongation at 60 °C for 1 min. All samples were run in parallels. The reactions were carried out in Applied Biosystems Viia7 qRT-PCR (Life technologies).

### Direct and indirect droplet digital PCR on cfDNA in plasma

To assess primer concentration and to test a possible direct quantification of cfDNA in plasma, two different primer pairs, *HK2* for ncfDNA and *NADH6* for mtcfDNA, were analyzed in plasma samples (direct) and in isolated cfDNA samples (indirect) using ddPCR. Samples for direct ddPCR were simply centrifuged for 10 min at 4 °C and 1,600 × g before being added to the reaction mix.

An initial reaction pool containing 11 µl 2 × QX200 ddPCR EvaGreen Supermix, 10 µl template, and 1 µl primer mix (100, 150, or 200 nM) was thoroughly mixed before transferring into 20 µl reaction mix and 70 µl Droplet Generation Oil for EvaGreen® to sample- and oil wells in a DG8™ Cartridge for QX200™ Droplet Generator (all ddPCR instruments and chemical were obtained from BioRad) respectively, then covered with Droplet Generator DG8™ Gasket. Droplets were generated using QX200 Droplet Generator, then ~ 40 µl droplets were transferred to a 96-Well ddPCR plate semi skirted and heat-sealed with Pierceable Foil Heat Seal in a PX1 plate sealer in 180 °C for 5 s before thermal cycling was performed on a Veriti™ 96-well Thermal Cycler (Applied Biosystems). Samples were analyzed on QX200 Droplet Reader with QuantaSoft Software.

Based on the primer assessment, extracted cfDNA from different timepoints and treatment groups were quantified using ddPCR. Reaction mixes containing 11 µl EvaGreen, 6.6 µl primermix (200 nM), and 4.4 µl cfDNA were prepared and analysed as previously described.

### Data provision

All clinical, qRT-PCR, and ddPCR raw data are provided (Supplementary files).

### Statistical analysis

Statistical analysis was performed using GraphPad Prism 9 (GraphPad Prism Software Inc.). The normality assumption was assessed by Shapiro–Wilk normality test and quantile–quantile (Q-Q)-plot. Paired t-test was applied to normally distributed data and expressed as mean ± standard deviation (S.D.), while a non-parametric test, the Wilcoxon test, was applied if normal distribution criteria were not met. P-values were corrected for multiple tests using the Bonferroni method. P < 0.008 for qRT-PCR and p < 0.0125 for ddPCR results were accepted as statistically significant.

**Fig. 2:**
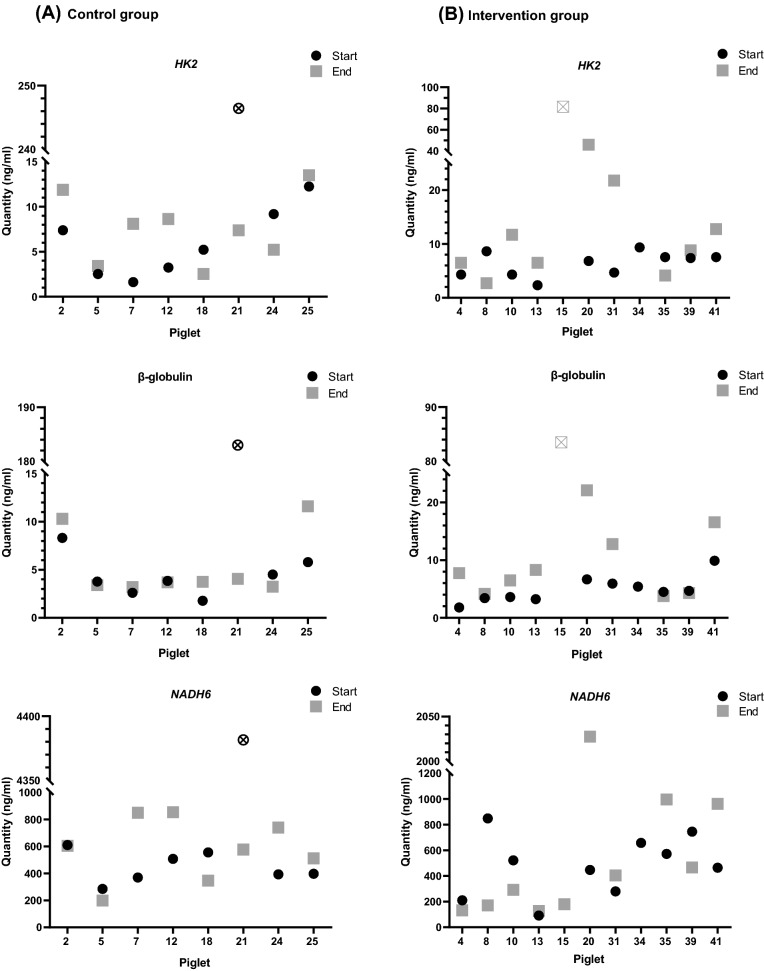
Concentrations of nuclear and mitochondrial cfDNA in plasma samples of the intervention versus the control group, measured by qRT-PCR. The amount of cfDNA in the plasma of newborn piglets in **A**. the control or **B**. the intervention group was determined at baseline (= start or prior to hypoxia) and at the end of hypoxia (for the sham-operated control group at 40–45 min, respectively). NcfDNA was analyzed at two nuclear loci, *HK2* and β-globulin, and one mitochondrial locus *NADH6* using qRT-PCR. (Outlier samples, identified by the IQR method are indicated with ⊗ or ☒)

Outliers identification was performed with the interquartile range (IQR) method. The IQR is defined as the difference between the third (Q3) and the first (Q1) quartile. The normal data range was defined with lower limit as Q1− 1.5*IQR and upper limit as Q3 + 1.5*IQR. Any data point outside this range was considered as an outlier and replaced with the median value of the respective group in the downstream analysis. Outliers were detected (Figs. 2. and 4.) based on statistical, technical, or biological criteria: if the data point was an outlier with both methods (qRT-PCR and ddPCR) and/or the animal was flagged having abnormal organs or died before the end of the experiment. A Bonferroni correction was performed where applicable.

## Results

### The piglet model of perinatal asphyxia

The clinical characteristics of the piglets were estimated by measuring Hb levels, BE, and MABP in the control- and in the intervention group. Previous studies have demonstrated that a lack of oxygen in the developing brain of a neonate causes cellular energy failure, generation of ROS, accumulation of hypoxanthine and lactate, decreased pH, and damages to the cell structures [34]. Piglets in the intervention group revealed significantly higher BE levels at the end of hypoxia (p < 0.0001) and at the end of the study (p = 0.030), further significantly lower MABP values (p < 0.0001) at the end of hypoxia in comparison to the sham-operated control group, which were not exposd to hypoxia (Table 1. and Supplementary Table 1.).

**Table 1:** Clinical characteristics were determined by measuring Hb levels, BE, and MABP in control and intervention group (median values)

Clinical values (median)		Control group	Intervention group	p-value
Hb (g/ml)	Start “hypoxia”	8.0	7.6	0.592
	End “hypoxia”	7.8	7.6	0.906
	End study	7.6	6.2	0.351
BE (mM)	Start “hypoxia”	1.2	1.8	0.474
	End “hypoxia”	0.6	-20.7	** < *0.0001***
	End study	1.6	-11.7	***0.030***
MABP (mmHg)	Start “hypoxia”	58.0	54.0	0.516
	End “hypoxia”	53.5	22.2	** < *0.0001***
	End study	55.5	29.0	0.083

*Hb* hemoglobin; *BE* base excess; and *MABP* mean arterial blood pressure. Clinical values ​​that were significantly different (p-value < 0.05) between the control and the intervention groups are marked in bold

### Yield and fragment size of cfDNA

The yield and fragment size of extracted cfDNA from the original and modified isolation protocols were estimated (Supplement Table 2.). The average fragment size of the first two aliquots was 240 bp but the cfDNA concentrations were outside the functional range for the assay. Using the modified protocol for the next three aliquots, we gained approximately three-fold higher average cfDNA concentrations within the functional range of the assay. The average fragment size remained at 219 bp. Further, we estimated the influence of freezing–thawing cycles to the length of the cfDNA fragments using the Agilent cfDNA Screen Tape. Changes and distributions in fragment size (p = 0.499) and concentration (p = 0.530) of cfDNA after five freeze–thaw cycles were < 5% and consequently not influencing the overall results (Supplementary Table 2.).

### Estimating the differences in cfDNA concentrations based on different genomic loci, cellular origins, and intervention procedures applying the qRT-PCR method

#### Comparison of the levels of ncfDNA determined at two different loci by qRT-PCR

To estimate the effect of different nuclear locations on the amounts of ncfDNA in plasma, samples of piglets exposed to hypoxia (intervention) versus sham-operated controls were determined at two different nuclear loci, *HK2* and β-globulin, respectively. The differences between the baseline and the end of hypoxia cfDNA concentrations were not statistically significant in any of the groups. Nevertheless, the end-point ncfDNA amount in samples of piglets exposed to hypoxia revealed an increasing trend of ncfDNA quantities compared to baseline values with both primer pairs (median): 7.1 ng/ml to 9.5 ng/ml for *HK2*, and 4.6 ng/ml to 7.9 ng/ml for β-globulin, respectively (Fig. 2.). For the *HK2* primers, we observed the same tendency in the levels of ncfDNA, even in the sham-operated control group; 5.8 ng/ml to 7.7 ng/ml, respectively. Sham-operated piglets revealed similar medians of baseline and end of hypoxia values using β-globulin primers: 4.0 ng/ml and 3.7 ng/ml, respectively.

#### Comparison of the concentrations of the levels of ncfDNA versus mtcfDNA using qRT-PCR

**Fig. 3:**
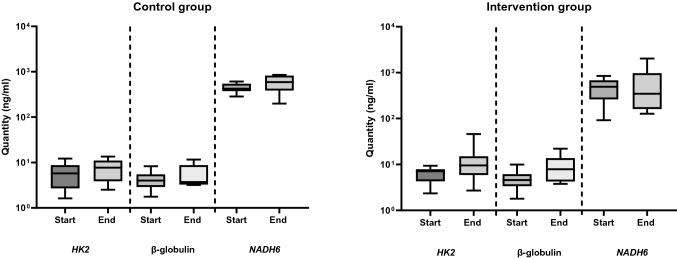
CfDNA concentrations, measured in plasma samples of newborn piglets exposed to hypoxia versus the control group, using the qRT-PCR method. Three different primer pairs were used for cfDNA assessment: *HK2* and β-globulin for ncfDNA and *NADH6* for mtcfDNA. Concentration of ncfDNA and mtcfDNA were determined in the sham-operated control and in the intervention group at two different time points (baseline (= start) and at the end of hypoxia or after 40–45 min for the sham-operated control group, respectively). An increasing trend in cfDNA concentrations was observed at the end of hypoxia

It is still a matter of debate, if the various diseases and conditions leading to an increase in cfDNA levels are due to changes in ncfDNA, mtcfDNA, or both. To this end, we quantified the amounts of ncfDNA and mtcfDNA at baseline and at the end of hypoxia for the intervention versus the control group. MtcfDNA concentrations were approximately 100-fold higher in comparison to the levels of ncfDNA (Fig. 3. and Supplementary Table 3.a. and b.). We observed a decreasing trend in the amount of cfDNA at the end of hypoxia compared to the baseline in the intervention group: 492.8 ng/ml to 347.9 ng/ml. Furthermore, the control group revealed an increasing trend in cfDNA concentrations at the end of hypoxia, 425.0 ng/ml to 590.6 ng/ml, respectively (Fig. 3. and Supplementary Table 3.a. and b.).

### Estimating differences of ncfDNA and mtcfDNA concentrations in the intervention and control group determined by ddPCR

**Fig. 4:**
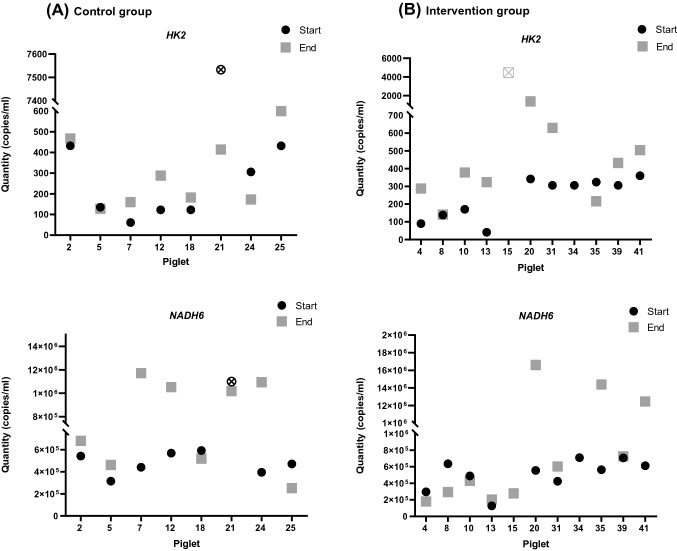
Levels of cfDNA in the intervention and the control group determined by ddPCR. The quantities of ncfDNA and mtcfDNA in samples of the intervention and the sham-operated control group were determined at the baseline (= start) and at the end of hypoxia or at 40–45 min for the sham-operated control group using ddPCR. (Outlier samples, identified by the IQR method are indicated with ⊗ or ☒)

**Fig. 5:**
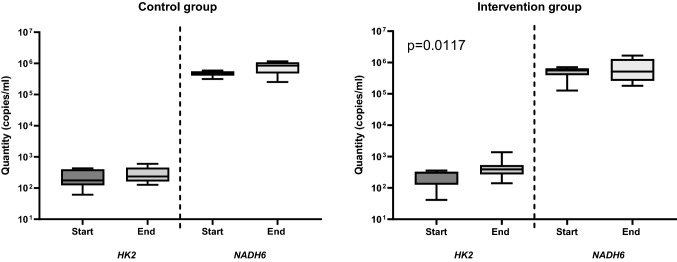
Comparison of ncfDNA versus mtcfDNA levels in newborn piglets exposed to hypoxia or in the control group using ddPCR. For quantification of cfDNA two primer pairs were used: *HK2* for ncfDNA and *NADH6* for mtcfDNA. The levels of ncfDNA and mtcfDNA were assessed at the baseline (= start) and at the end of hypoxia (or at 40–45 min for the sham-operated control group, respectively) in piglets of the intervention or the sham-operated control group. A statistically significant (p = 0.0117) increase in median ncfDNA concentration was observed at the end of hypoxia in the intervention group

Measuring cfDNA using qRT-PCR is an easy, fast, and cheap approach; however, in particular for samples with low amounts of cfDNA the more exact but technically demanding state-of-the art technique seems preferable. Using the ddPCR technique, the quantity of ncfDNA was higher at the end of hypoxia in comparison to its baseline values from (median) 177.8 to 234.9 copies/ml in the control group and 306.0 to 391.5 copies/ml in the intervention group, respectively (Fig. 4., Fig. 5., and Supplementary Table 3.a. and b.). The difference between baseline and end ncfDNA concentrations were only statistically significant in the intervention group (p = 0.0117). MtcfDNA levels resulted in > 1000-fold higher levels than ncfDNA (Fig. 4., Fig. 5., and Supplementary Table 3.a. and b.). We observed a decrease from 558,900 to 516,420 copies/ml in the intervention group and an increase from 488,745 to 849,600 copies/ml in mtcfDNA levels at the end of hypoxia in the control group. In an attempt to directly quantify cfDNA in plasma samples, *HK2* and *NADH6* were analysed in diluted and undiluted plasma samples using ddPCR; however, the cfDNA levels in the direct method were below the detection limit.

## Discussion

Previous studies revealed an emerging prospect of cfDNA as a novel biomarker in numerous fields of medicine, including oncology, transplantation medicine, and cardiovascular diseases [35]. Quantification of cfDNA offers tremendous potential as liquid biopsy applications with many advantages in comparison to traditional tissue biopsies: it is easy to obtain, minimally invasive, and suitable for serial monitoring allowing the assessment of disease progression and response to treatment [36, 37]. However, using circulating cfNAs as a biomarker for diagnosis or monitoring of the progression of a disease may only be proposed, if sensitive and reliable methods for the assessment of cfDNA are available. Quantification of low-copy DNA is limited not only by the assay performance characteristics, but also by the stochastic effects associated with low copy numbers and sub-sampling, which may contribute to measurement uncertainties at low limits of detection [38]. The majority of available methods evaluating nucleic acids concentrations are based on absorbance, fluorometric measurement, and gel electrophoresis; however, all of them exhibit some sort of inaccuracies. The qRT-PCR technique is a well-established method, relatively cheap, easy to analyze, and time-efficient; but a general lack of standardization and the relatively low precision have compromised its utility [4].

The novel, state-of-the-art method digital PCR provides highly accurate and direct quantification of nucleic acids without the need for extrapolation, standard curve, or references [39]. The advantage of digital PCR comprehends that the results are directly provided as the number of copies of the target molecule per µl of reaction and it has a higher precision and sensitivity in detecting low target copies. Further, digital PCR is also relatively insensitive to potential PCR inhibitors, resisting variations of qRT-PCR efficiencies [4, 38–41].

Here in this study, we compared the distribution of ncfDNA and mtcfDNA levels detected by qRT-PCR versus ddPCR. The results indicate that the temporal distribution of nuclear and mitochondrial cfDNA amount obtained by both methods reveal similar trends. However, in our study, a statistically significant (p = 0.0017) increase in the amount of ncfDNA at the end of hypoxia was only observed with the ddPCR method, indicating a potential benefit of this more exact method to detect a physiologic response that is only minimal.

Further, we compared the relative changes in the amount of cfDNA for both, the control and the intervention group. Again, the results showed comparable variations for both instruments. Even though we started with small starting volumes (450 μl or less), the qRT-PCR method for cfDNA quantification was able to detect the small amount of these molecules and its results were comparable to ddPCR. On the other hand, a high amount of mtcfDNA presented a challenge for ddPCR and may cause ambiguous numbers.

Besides the methodological challenges, many unresolved questions regarding the structure and origin of cfDNA remain (Fig. 1.). The general term of circulating cfDNA comprehends free DNA fragments, vesicle-bound DNA, and macromolecular DNA complexes [42]. Free DNA fragments are cleaved by DNases in blood until they are completely lost in body fluids, whereas circulating nucleosomes are protected from degradation [42]. Different cell types have diverse nucleosome positioning [43], which may determine the accessibility of cfDNAs to nucleases and thus, may play a role in generating cleavage patterns [44].

In this study, we investigated the fragment size of cfDNA and determined it to an average of 219 bp, which is somehow higher than reported elsewhere. One could speculate, if the longer size is due to contamination from genomic DNA. However, this is counteracted by the various precautions taken to ensure high cfDNA yield, including the preference of the extraction method with specific affinity for short DNA fragments and the choice of primer pairs resulting in short PCR products. Further, we previously performed control experiments using electrophoresis and we tested different primer sets generating long or short PCR products to verify our approach [13].

We investigated two distinct different genomic loci of ncfDNA, *HK2* and β-globulin, together with the temporal distribution of cfDNA by applying the qRT-PCR method. Our results indicate that the mean concentrations of the two target cfDNA molecules detected in plasma are comparable; however, we observed an evident increase in the amount of cfDNA at the β-globulin loci at the end of hypoxia compared to the baseline in the intervention group. Related results towards elevated cfDNA levels were observed using the *HK2* primers. Despite the similar trends of the two genomic loci we investigated, one may consider that nucleosome positioning, among other factors (e.g., cancer specific mutations and/or up-regulation of specific genes in different clinical settings), may influence the amount of detectable cfDNA.

Both, nuclear and mitochondrial cfDNA are attracting more and more attention in different fields of clinical practice [19, 32, 35, 45]. Despite the increasing number of published studies investigating cfDNA, there has not been much emphasis on exploring nuclear and mitochondrial cfDNA separately. It is still unclear, which of the two sources might be more relevant for the prediction and monitoring of a disease or a condition, as some studies have reported contradictory results [20–22]. NcfDNA in the plasma of healthy humans is primarily of lymphoid and myeloid origin [46], which is consistent with the finding that the major source of cfDNA is the apoptosis of hematopoietic cells [42]. On the other hand, patients with different diseases (e.g., cancer, stroke, autoimmune disorders, and others) have additional contributions of cfDNAs from non-hematopoietic tissues [43, 47]. Mitochondria carry multiple copies of their circular genome, approximately 50,000 fold in the plasma of healthy individuals [47]. Different structures of mtcfDNA are present in the circulation: extruded whole mitochondria, mtDNA within vesicles and microparticles, protein-bound mtDNA, exposed cell-free mtDNA fragments, and intact mitochondrial genomes [47, 48]. Recent studies suggest that most of the mtcfDNA exists as whole mitochondria or it is encapsulated within the vesicles [42]. Since mtcfDNA is a small molecule not protected by histones, mtcfDNA fragments are in general smaller in comparison to the ncfDNA structures [42]. Beside their role in energy production, the mitochondria participate in production and decomposition of ROS, cell death, and different cell signaling pathways [47]. Studies showed that the different types of cells (e.g., platelets, leukocytes, astrocytes, and immortalized cell lines) can actively release mtcfDNA [46]. Consequently, increase in mtcfDNA levels might be a consequence of various types of stress responses and—similar to ncfDNA—both, passive (apoptosis, necrosis, NETosis, and mitophagy) and active processes of mtcfDNA release have been proposed closely associated with the generation of ROS [49]. Further, it remains unresolved, whether ncfDNA or mtcfDNA is a more sensitive marker for oxidative stress reactions in a cell [13]. We amplified nuclear and mitochondrial targets and compared their values. Our results revealed that mtcfDNA levels are approximately 100 to 1,000-fold higher (depending on the quantification method) than ncfDNA, in the control and in the intervention group. Additionally, mtcfDNA displayed an opposite trend compared to ncfDNA, supporting the hypothesis that their mechanism of release and their role differ.

### Limitations

The main limitations of our study were the small sample number of investigated piglets and the unavailability of some of the plasma samples, which might have altered the statistical power of the analyses. We compared only two time points in a period of only 60 min. However, the piglets were observed for another 9.5 h and their samples were taken periodically. It would be interesting to investigate the amount of cfDNA over a longer period and compare the results to the clinical status of the piglets. Further, the volumes of plasma samples were scarce, 450 μl or less, which may influence the quantity of cfDNA. We compared only the trends of cfDNA concentration changes and not absolute values since the results obtained by qRT-PCR and ddPCR are given in different units (ng/ml versus copies/ml).

## Conclusion

We explored different genomic loci of ncfDNA and differences between ncfDNA versus mtcfDNA quantities and their temporal distribution from piglets’ plasma using samples from a newborn model of asphyxia. We found an increasing trend in the levels of ncfDNA in piglets exposed to hypoxia compared to control at the end of hypoxia. In addition, median quantity of mtcfDNA was approximately 100 to 1,000-fold higher than that of ncfDNA. Comparing the qRT-PCR versus the ddPCR technique, we close that—despite similar patterns—the more sensitive and precise ddPCR method seems beneficial due to its higher affinity to small changes in cfNAs.

## Supplementary Information

Below is the link to the electronic supplementary material.

Supplementary file1 (PDF 67 KB)

Supplementary file2 (XLSX 111 KB)

Supplementary file3 (XLSX 17 KB)

Supplementary file4 (PDF 114 KB)

Supplementary file5 (PDF 155 KB)

## References

[1] Aucamp J, Bronkhorst AJ, Badenhorst CPS, Pretorius PJ (2018). The diverse origins of circulating cell-free DNA in the human body: a critical re-evaluation of the literature: the diverse origins of circulating cell-free DNA. Biol Rev.

[2] Bronkhorst AJ, Ungerer V, Holdenrieder S (2019). The emerging role of cell-free DNA as a molecular marker for cancer management. Biomol Detect Quantif.

[3] Pös O, Biró O, Szemes T, Nagy B (2018). Circulating cell-free nucleic acids: characteristics and applications. Eur J Hum Genet.

[4] Ranucci R, Casadio V, Salvi S (2019). Cell-Free DNA: Applications in Different Diseases. Cell-free DNA as Diagnostic Markers Methods in Molecular Biology.

[5] Szilágyi M, Pös O, Márton É, Buglyó G, Soltész B, Keserű J, Penyige A, Szemes T, Nagy B (2020). Circulating cell-free nucleic acids: main characteristics and clinical application. Int J Mol Sci.

[6] Grabuschnig S, Bronkhorst AJ, Holdenrieder S, Rosales RI, Schliep KP, Schwendenwein D, Ungerer V, Sensen CW (2020). Putative origins of cell-free DNA in humans: a review of active and passive nucleic acid release mechanisms. Int J Mol Sci.

[7] Kustanovich A, Schwartz R, Peretz T, Grinshpun A (2019). Life and death of circulating cell-free DNA. Cancer Biol Ther.

[8] Gomez-Lopez N, Romero R, Schwenkel G, Garcia-Flores V, Panaitescu B, Varrey A, Ayoub F, Hassan SS, Phillippe M (2020). Cell-free fetal DNA increases prior to labor at term and in a subset of preterm births. Reprod Sci.

[9] Czeiger D, Shaked G, Eini H, Vered I, Belochitski O, Avriel A, Ariad S, Douvdevani A (2011). Measurement of circulating cell-free DNA levels by a new simple fluorescent test in patients with primary colorectal cancer. Am J Clin Pathol.

[10] Breitbach S (2014). Direct quantification of cell-free, circulating DNA from unpurified plasma. Ed T Gilbert PLoS ONE.

[11] Wu T-L, Zhang D, Chia J-H, Tsao K-C, Sun C-F, Wu JT (2002). Cell-free DNA: measurement in various carcinomas and establishment of normal reference range. Clin Chim Acta.

[12] Manueldas S (2018). Temporal patterns of circulating cell-free DNA (cfDNA) in a newborn piglet model of perinatal asphyxia Ed L.G. Koniaris. PLOS ONE.

[13] Rajar P, Åsegg-Atneosen M, Saugstad OD, Solberg R, Baumbusch LO (2019). Quantification of circulating cell-free DNA (cfDNA) in urine using a newborn piglet model of asphyxia Ed M. Antopolsky. PLOS ONE.

[14] Fritz JV, Heintz-Buschart A, Ghosal A, Wampach L, Etheridge A, Galas D, Wilmes P (2016). Sources and functions of extracellular small RNAs in human circulation. Annu Rev Nutr.

[15] Kubiritova Z, Radvanszky J, Gardlik R (2019). Cell-free nucleic acids and their emerging role in the pathogenesis and clinical management of inflammatory bowel disease. Int J Mol Sci.

[16] el Andaloussi S, Mäger I, Breakefield XO, Wood MJA (2013). Extracellular vesicles: biology and emerging therapeutic opportunities. Nat Rev Drug Discov.

[17] Pös Z, Pös O, Styk J, Mocova A, Strieskova L, Budis J, Kadasi L, Radvanszky J, Szemes T (2020). Technical and methodological aspects of cell-free nucleic acids analyzes. Int J Mol Sci.

[18] Ventura W, Nazario-Redondo C, Sekizawa A (2013). Non-invasive prenatal diagnosis from the perspective of a low-resource country. Int J Gynaecol Obstet.

[19] Soltész B (2019). Quantification of peripheral whole blood, cell-free plasma and exosome encapsulated mitochondrial DNA copy numbers in patients with atrial fibrillation. J Biotechnol.

[20] Mouliere F, Thierry AR (2012). The importance of examining the proportion of circulating DNA originating from tumor, microenvironment and normal cells in colorectal cancer patients. Expert Opin Biol Ther.

[21] Gaziev A, Abdullaev S, Minkabirova G, Kamenskikh K (2016). X-rays and metformin cause increased urinary excretion of cell-free nuclear and mitochondrial DNA in aged rats. J Circ Biomark.

[22] Kohler C (2009). Levels of plasma circulating cell free nuclear and mitochondrial DNA as potential biomarkers for breast tumors. Mol Cancer.

[23] Stortz JA (2019). Cell-free nuclear, but not mitochondrial, DNA concentrations correlate with the early host inflammatory response after severe trauma. Sci Rep.

[24] Greco P (2020). Pathophysiology of hypoxic–ischemic encephalopathy: a review of the past and a view on the future. Acta Neurol Belg.

[25] Endrich O, Rimle C, Zwahlen M, Triep K, Raio L, Nelle M (2017). Asphyxia in the newborn: evaluating the accuracy of ICD coding, clinical diagnosis and reimbursement: observational study at a swiss tertiary care center on routinely collected health data from 2012–2015. Ed U. Simeoni PLOS ONE.

[26] Tuaeva NO, Abramova ZI, Sofronov VV (2008). The origin of elevated levels of circulating DNA in blood plasma of premature neonates. Ann N Y Acad Sci.

[27] Martignano F, Casadio V, Salvi S (2019). Cell-Free DNA: An Overview of Sample Types and Isolation Procedures. Cell-free DNA as Diagnostic Markers Methods in Molecular Biology.

[28] Zhao Y, Li Y, Chen P, Li S, Luo J, Xia H (2019). Performance comparison of blood collection tubes as liquid biopsy storage system for minimizing cfDNA contamination from genomic DNA. J Clin Lab Anal.

[29] Medina Diaz I, Nocon A, Mehnert DH, Fredebohm J, Diehl F, Holtrup F (2016). Performance of streck cfDNA blood collection tubes for liquid biopsy testing. Ed K.Y.K. Chan PLOS ONE.

[30] Grölz D, Hauch S, Schlumpberger M, Guenther K, Voss T, Sprenger-Haussels M, Oelmüller U (2018). Liquid Biopsy preservation solutions for standardized pre-analytical workflows—venous whole blood and plasma. Curr Pathobiol Rep.

[31] Pérez-Barrios C (2016). Comparison of methods for circulating cell-free DNA isolation using blood from cancer patients: impact on biomarker testing. Transl Lung Cancer Res.

[32] Sanchez C, Snyder MW, Tanos R, Shendure J, Thierry AR (2018). New insights into structural features and optimal detection of circulating tumor DNA determined by single-strand DNA analysis. Npj Genomic Med.

[33] Benterud T, Pankratov L, Solberg R, Bolstad N, Skinningsrud A, Baumbusch L, Sandvik L, Saugstad OD (2015). Perinatal asphyxia may influence the level of beta-amyloid (1–42) in cerebrospinal fluid: an experimental study on newborn pigs. Ed T Raju PLOS ONE.

[34] Saugstad OD, Sejersted Y, Solberg R, Wollen EJ, Bjørås M (2012). Oxygenation of the newborn: a molecular approach. Neonatology.

[35] Fernández-Lázaro D, García Hernández JL, García AC, Córdova MA, Mielgo-Ayuso J, Cruz-Hernández JJ (2020). Liquid biopsy as novel tool in precision medicine: origins, properties, identification and clinical perspective of cancer’s biomarkers. Diagnostics.

[36] Ulz P (2019). Inference of transcription factor binding from cell-free DNA enables tumor subtype prediction and early detection. Nat Commun.

[37] Poulet G, Massias J, Taly V (2019). Liquid biopsy: general concepts. Acta Cytol.

[38] Szpechcinski A (2015). Cell-free DNA levels in plasma of patients with non-small-cell lung cancer and inflammatory lung disease. Br J Cancer.

[39] Campomenosi P, Gini E, Noonan DM, Poli A, D’Antona P, Rotolo N, Dominioni L, Imperatori A (2016). A comparison between quantitative PCR and droplet digital PCR technologies for circulating microRNA quantification in human lung cancer. BMC Biotechnol.

[40] Devonshire AS, Whale AS, Gutteridge A, Jones G, Cowen S, Foy CA, Huggett JF (2014). Towards standardisation of cell-free DNA measurement in plasma: controls for extraction efficiency, fragment size bias and quantification. Anal Bioanal Chem.

[41] Hayden RT, Gu Z, Ingersoll J, Abdul-Ali D, Shi L, Pounds S, Caliendo AM (2013). Comparison of droplet digital PCR to real-time PCR for quantitative detection of cytomegalovirus. J Clin Microbiol.

[42] Dache AA, Z. (2020). Blood contains circulating cell-free respiratory competent mitochondria. FASEB J.

[43] Zhu G, Ye X, Dong Z, Lu YC, Sun Y, Liu Y, McCormack R, Gu Y, Liu X (2015). Highly sensitive droplet digital PCR method for detection of EGFR-activating mutations in plasma cell-free DNA from patients with advanced non-small cell lung cancer. J Mol Diagn.

[44] Keller L, Belloum Y, Wikman H, Pantel K (2021). Clinical relevance of blood-based ctDNA analysis: mutation detection and beyond. Br J Cancer.

[45] Vrablicova Z, Tomova K, Tothova L, Babickova J, Gromova B, Konecna B, Liptak R, Hlavaty T, Gardlik R (2020). Nuclear and mitochondrial circulating cell-free DNA is increased in patients with inflammatory bowel disease in clinical remission. Front Med.

[46] Duque-Afonso J, Waterhouse M, Pfeifer D, Follo M, Duyster J, Bertz H, Finke J (2018). Cell-free DNA characteristics and chimerism analysis in patients after allogeneic cell transplantation. Clin Biochem.

[47] Mouliere F, Robert B, Arnau PE, Del Rio M, Ychou M, Molina F, Gongora C, Thierry AR (2011). High Fragmentation characterizes tumour-derived circulating DNA. Ed T Lee PLoS ONE.

[48] Thurairajah K, Briggs GD, Balogh ZJ (2018). The source of cell-free mitochondrial DNA in trauma and potential therapeutic strategies. Eur J Trauma Emerg Surg.

[49] Sansone P (2017). Packaging and transfer of mitochondrial DNA via exosomes regulate escape from dormancy in hormonal therapy-resistant breast cancer. Proc Natl Acad Sci.

