# Supplementary material for: Assessing nuclear versus mitochondrial cell-free DNA (cfDNA) by qRT-PCR and droplet digital PCR using a piglet model of perinatal asphyxia
Source: Mol Biol Rep. 2022 Dec 13;50(2):1533–44. doi: 10.1007/s11033-022-08135-0 (PMC9889441; doi:10.1007/s11033-022-08135-0)
Supplement: Supplementary file 1 — Supplementary file1 (PDF 67 KB) [file 11033_2022_8135_MOESM1_ESM.pdf]

Supplementary to

"Assessing nuclear versus mitochondrial cell-free DNA (cfDNA) by qRT-PCR and droplet digital PCR using a piglet model of perinatal asphyxia" published in Molecular Biology reports by Marie Bitenc, Benedicte Grebstad Tune, Maria Melheim, Monica Atneosen-Åsegg, Xiaoran Lai, Polona Rajar, Rønnaug Solberg, and Lars Oliver Baumbusch at the Department of Pediatric Research, Division of Paediatric and Adolescent Medicine, Oslo University Hospital Rikshospitalet, Oslo, Norway. Email: lars.o.baumbusch@rr-research.no.

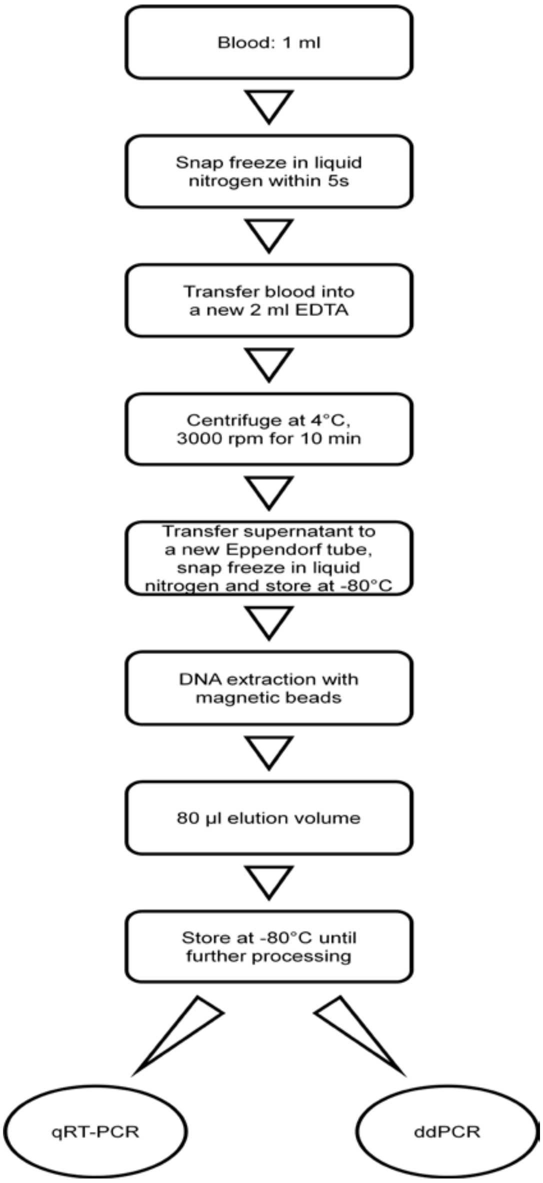

Supplementary figure 1. Flow chart for cfDNA extraction and quantification. Schematic presentation of the method tested for cell-free DNA (cfDNA) assessment in plasma samples. For cfDNA quantification, all samples were measured by A. qRT-PCR or B. ddPCR (droplet digital PCR), with primers detecting nuclear (ncfDNA) and mitochondrial cfDNA (mtcfDNA).
