# Supplementary material for: Assessing nuclear versus mitochondrial cell-free DNA (cfDNA) by qRT-PCR and droplet digital PCR using a piglet model of perinatal asphyxia
Source: Mol Biol Rep. 2022 Dec 13;50(2):1533–44. doi: 10.1007/s11033-022-08135-0 (PMC9889441; doi:10.1007/s11033-022-08135-0)
Supplement: Supplementary file 4 — Supplementary file4 (PDF 114 KB) [file 11033_2022_8135_MOESM4_ESM.pdf]

Supplementary to

"Assessing nuclear versus mitochondrial cell-free DNA (cfDNA) by qRT-PCR and droplet digital PCR using a piglet model of perinatal asphyxia" published in Molecular Biology reports by Marie Bitenc, Benedicte Grebstad Tune, Maria Melheim. Monica Atneosen-Åsegg, Xiaoran Lai, Polona Rajar, Rønnaug Solberg, and Lars Oliver Baumbusch at the Department of Pediatric Research, Division of Paediatric and Adolescent Medicine, Oslo University Hospital Rikshospitalet, Oslo, Norway. Email: lars.o.baumbusch@rr-research.no.

Supplementary table 2. Fragment size and cfDNA yield. Average size, cfDNA concentration, region molarity, and the cfDNA percentage of total DNA are presented.

<table>
<tr><th colspan="13">Measurement of cfDNA fragment size and yield</th></tr>
<tr><th>Sample description</th><th colspan="3">Average size (bp)</th><th colspan="3">Conc. (pg/µl)</th><th colspan="3">Total DNA (%)</th><th colspan="3">Region molarity (pmol/l)</th></tr>
<tr><td>Pooled sample 1</td><td colspan="3">253</td><td colspan="3">20.1</td><td colspan="3">68.33</td><td colspan="3">220</td></tr>
<tr><td>Pooled sample 2</td><td colspan="3">227</td><td colspan="3">22.8</td><td colspan="3">72.64</td><td colspan="3">256</td></tr>
<tr><td>Pooled sample 3</td><td colspan="3">218</td><td colspan="3">96.9</td><td colspan="3">91.23</td><td colspan="3">907</td></tr>
<tr><td>Pooled sample 4</td><td colspan="3">219</td><td colspan="3">62.9</td><td colspan="3">92.76</td><td colspan="3">587</td></tr>
<tr><td>Pooled sample 5</td><td colspan="3">221</td><td colspan="3">72.2</td><td colspan="3">89.59</td><td colspan="3">625</td></tr>
<tr><th colspan="13">Freezing-thawing influence on cfDNA fragment length</th></tr>
<tr><th></th><th colspan="3">Average size (bp)</th><th colspan="3">Conc. (pg/µl)</th><th colspan="3">Total DNA (%)</th><th colspan="3">Difference in average size (%)</th></tr>
<tr><th>Piglet sample</th><th>12</th><th>13</th><th>14</th><th>12</th><th>13</th><th>14</th><th>12</th><th>13</th><th>14</th><th>12</th><th>13</th><th>14</th></tr>
<tr><td>Before 1st freeze</td><td>266</td><td>311</td><td>222</td><td>47.0</td><td>33.4</td><td>50.0</td><td>78.63</td><td>65.66</td><td>71.20</td><td colspan="3">N/A</td></tr>
<tr><td>2nd freeze</td><td>261</td><td>308</td><td>226</td><td>61.3</td><td>31.3</td><td>43.5</td><td>76.35</td><td>63.33</td><td>79.56</td><td>1.9</td><td>1.0</td><td>1.8</td></tr>
<tr><td>3rd freeze</td><td>266</td><td>298</td><td>226</td><td>56.9</td><td>31.8</td><td>32.1</td><td>74.51</td><td>66.05</td><td>87.87</td><td>0.0</td><td>4.2</td><td>1.8</td></tr>
<tr><td>4th freeze</td><td>265</td><td>297</td><td>230</td><td>43.4</td><td>25.9</td><td>42.3</td><td>77.33</td><td>61.97</td><td>81.49</td><td>0.4</td><td>4.5</td><td>3.6</td></tr>
<tr><td>5th freeze</td><td>257</td><td>298</td><td>229</td><td>50.0</td><td>30.7</td><td>43.4</td><td>73.85</td><td>63.3</td><td>78.18</td><td>3.4</td><td>4.2</td><td>3.2</td></tr>
<tr><td>Before 1st freeze vs 5th freeze (T-test)</td><td colspan="3">P=0.499</td><td colspan="3">P=0.530</td><td colspan="6">N/A</td></tr>
</table>

Measurement of cfDNA fragment size and yield

Sample description	Average size (bp)	Conc. (pg/µl)	Total DNA (%)	Region molarity (pmol/l)
Pooled sample 1	253	20.1	68.33	220
Pooled sample 2	227	22.8	72.64	256
Pooled sample 3	218	96.9	91.23	907
Pooled sample 4	219	62.9	92.76	587
Pooled sample 5	221	72.2	89.59	625

Freezing-thawing influence on cfDNA fragment length

	Average size (bp)			Conc. (pg/µl)			Total DNA (%)			Difference in average size (%)		
Piglet sample	**12**	**13**	**14**	**12**	**13**	**14**	**12**	**13**	**14**	**12**	**13**	**14**
Before 1st freeze	266	311	222	47.0	33.4	50.0	78.63	65.66	71.20	N/A		
2nd freeze	261	308	226	61.3	31.3	43.5	76.35	63.33	79.56	1.9	1.0	1.8
3rd freeze	266	298	226	56.9	31.8	32.1	74.51	66.05	87.87	0.0	4.2	1.8
4th freeze	265	297	230	43.4	25.9	42.3	77.33	61.97	81.49	0.4	4.5	3.6
5th freeze	257	298	229	50.0	30.7	43.4	73.85	63.3	78.18	3.4	4.2	3.2
Before 1st freeze vs 5th freeze (T-test)	P=0.499			P=0.530			N/A					
