# Supplementary material for: Assessing nuclear versus mitochondrial cell-free DNA (cfDNA) by qRT-PCR and droplet digital PCR using a piglet model of perinatal asphyxia
Source: Mol Biol Rep. 2022 Dec 13;50(2):1533–44. doi: 10.1007/s11033-022-08135-0 (PMC9889441; doi:10.1007/s11033-022-08135-0)
Supplement: Supplementary file 5 — Supplementary file5 (PDF 155 KB) [file 11033_2022_8135_MOESM5_ESM.pdf]

Supplementary to

"Assessing nuclear versus mitochondrial cell-free DNA (cfDNA) by qRT-PCR and droplet digital PCR using a piglet model of perinatal asphyxia" published in Molecular Biology reports by Marie Bitenc, Benedicte Grebstad Tune, Maria Melheim, Monica Atneosen-Åsegg, Xiaoran Lai, Polona Rajar, Rønnaug Solberg, and Lars Oliver Baumbusch at the Department of Pediatric Research, Division of Paediatric and Adolescent Medicine, Oslo University Hospital Rikshospitalet, Oslo, Norway. Email: lars.o.baumbusch@rr-research.no.

Supplementary table 3.a. qRT-PCR versus ddPCR. cfDNA concentration at baseline and at the end of hypoxia in the plasma samples of newborn piglets in the control group and the intervention group (piglets exposed to hypoxia). Two different quantification methods were used to estimate cfDNA concentration, qRT-PCR and ddPCR. Three different primers were used for qRT-PCR: *HK2* (nuclear cfDNA), β-globulin (nuclear cfDNA), and *NADH6* (mitochondrial cfDNA). Two different primes were used for ddPCR: *HK2* (nuclear cfDNA) and *NADH6* (mitochondrial cfDNA).
LEGEND: NA - Some samples were not available due to limitation of the material.
* - These piglets were excluded.

Piglet No.	Control (C) or Intervention (I) group	NADH6				HK2				Beta globulin	
		qRT-PCR		ddPCR		qRT-PCR		ddPCR		qRT-PCR	
		Start concentration (ng/ml)	End concentration (ng/ml)	Start concentration (copies/ml)	End concentration (copies/ml)	Start concentration (ng/ml)	End concentration (ng/ml)	Start concentration (copies/ml)	End concentration (copies/ml)	Start concentration (ng/ml)	End concentration (ng/ml)
2	C	610.20	604.26	541800	680400	7.38	11.88	432	468	8.31	10.29
5	C	284.76	198.72	314460	461520	2.52	3.42	135	127.80	3.77	3.39
7	C	369.36	849.78	441180	1171800	1.62	8.1	61.20	160.20	2.60	3.21
12	C	508.14	854.28	568800	1053000	3.24	8.64	122.40	288	3.84	3.68
18*	C	555.48	345.78	592200	518940	5.22	2.52	122.40	181.80	1.77	3.75
21	C	4381.54	576.90	1100500	1018800	246.45	7.38	7533	414	183.02	4.05
24	C	392.94	739.98	394920	1094400	9.18	5.22	306	172.8	4.50	3.23
25	C	397.26	511.25	471060	251500	12.24	13.50	432	600	5.79	11.60
4*	I	210.06	132.12	297180	181080	4.32	6.48	90	288	1.80	7.74
8	I	848.52	170.82	635400	293580	8.64	2.70	138.6	140.4	3.42	4.14
10	I	521.64	291.42	489420	429840	4.32	11.70	171	378	3.60	6.48
13	I	91.80	127.62	126900	203580	2.34	6.48	41.4	324	3.24	8.28
15*	I	NA	179.28	NA	278820	NA	81.72	NA	4464	NA	83.52
20	I	446.76	2027.45	554400	1660600	6.84	46.00	342	1380	6.66	22.08
31	I	279.72	404.28	423900	603000	4.68	21.78	306	630	5.94	12.78
34*	I	657.72	NA	709200	NA	9.36	NA	306	NA	5.40	NA
35	I	571.86	995.94	563400	1438200	7.56	4.14	324	216	4.50	3.78
39	I	745.56	466.20	707400	725400	7.38	8.82	306	432	4.68	4.32
41*	I	464.04	961.74	612000	1245600	7.56	12.78	360	504	9.90	16.56

Supplementary table 3.b. Relative changes between end and start concentration obtained by qRT-PCR and ddPCR. Two different quantification methods (qRT-PCR and ddPCR) were used to estimate the cfDNA concentration in samples of piglets in the control or the intervention (piglets exposed to hypoxia) group. We used two different primers to compare the methods: A.) *HK2* (nuclear cfDNA) and B.) *NADH6* (mitochondrial cfDNA). The table shows means with standard deviation (SD) of relative changes in cfDNA concentrations (calculated by dividing end- with start cfDNA amount). Original values were included for every piglet sample. Piglet 15 and 34 were not involved in this analysis because only one time-point samples were available.

	qRT-PCR		ddPCR	
**Primer**	**Control group**	**Intervention group**	**Control group**	**Intervention group**
	**Mean (SD) of relative change**	**Mean (SD) of relative change**	**Mean (SD) of relative change**	**Mean (SD) of relative change**
HK2	1.6 (1.6)	2.5 (2.1)	1.3 (0.9)	2.6 (2.2)
NADH6	1.2 (0.7)	1.5 (1.3)	1.5 (0.8)	1.5 (0.9)
